# Abnormal functional connectivity during visuospatial processing is associated with disrupted organisation of white matter in autism

**DOI:** 10.3389/fnhum.2013.00434

**Published:** 2013-09-26

**Authors:** Jane McGrath, Katherine Johnson, Erik O'Hanlon, Hugh Garavan, Alexander Leemans, Louise Gallagher

**Affiliations:** ^1^Department of PsychiatryTrinity College Dublin, Ireland; ^2^Department of Psychology, University of Melbourne, MelbourneVictoria, Australia; ^3^Department of Psychiatry, Royal College of Surgeons in IrelandDublin, Ireland; ^4^Department of Psychology, University of Vermont, BurlingtonVT, USA; ^5^Image Sciences Institute, University Medical Centre UtrechtUtrecht, Netherlands

**Keywords:** neuroimaging, autism spectrum disorders, functional connectivity, diffusion tractography, constrained spherical deconvolution, visuospatial processing, structural connectivity, mental rotation

## Abstract

Disruption of structural and functional neural connectivity has been widely reported in Autism Spectrum Disorder (ASD) but there is a striking lack of research attempting to integrate analysis of functional and structural connectivity in the same study population, an approach that may provide key insights into the specific neurobiological underpinnings of altered functional connectivity in autism. The aims of this study were (1) to determine whether functional connectivity abnormalities were associated with structural abnormalities of white matter (WM) in ASD and (2) to examine the relationships between aberrant neural connectivity and behavior in ASD. Twenty-two individuals with ASD and 22 age, IQ-matched controls completed a high-angular-resolution diffusion MRI scan. Structural connectivity was analysed using constrained spherical deconvolution (CSD) based tractography. Regions for tractography were generated from the results of a previous study, in which 10 pairs of brain regions showed abnormal functional connectivity during visuospatial processing in ASD. WM tracts directly connected 5 of the 10 region pairs that showed abnormal functional connectivity; linking a region in the left occipital lobe (left BA19) and five paired regions: left caudate head, left caudate body, left uncus, left thalamus, and left cuneus. Measures of WM microstructural organization were extracted from these tracts. Fractional anisotropy (FA) reductions in the ASD group relative to controls were significant for WM connecting left BA19 to left caudate head and left BA19 to left thalamus. Using a multimodal imaging approach, this study has revealed aberrant WM microstructure in tracts that directly connect brain regions that are abnormally functionally connected in ASD. These results provide novel evidence to suggest that structural brain pathology may contribute (1) to abnormal functional connectivity and (2) to atypical visuospatial processing in ASD.

## Introduction

There is extensive evidence to suggest that autism is a disorder characterized by disrupted functional and structural neural connectivity. Abnormal inter- and intra-regional functional connectivity has been described whilst participants have performed various neuropsychological paradigms and whilst in the resting state. In parallel with functional connectivity research, a number of diffusion imaging studies in autism have demonstrated aberrant “structural connectivity”—a term referring to the integrity of white matter (WM) micro- and macrostructure. Abnormal functional connectivity between brain regions in autism may arise from disrupted organization of WM, but its pathophysiology is unknown. Despite the numerous studies that have consistently reported abnormal functional connectivity in autism, there is a surprising lack of research attempting to integrate analysis of functional and structural connectivity in the same study population, an approach that may provide key insights into the specific neurobiological underpinnings of altered functional connectivity in autism.

### Behavioral effects of disrupted neural connectivity in ASD

There appear to be significant behavioral effects of disrupted structural and functional connectivity in autism. In relation to structural connectivity, several studies have explored the correlation between WM organization and autism symptom severity. Fractional anisotropy (FA) is a widely used measure that provides information about the degree of WM organization. Increased severity of restricted and repetitive behaviors was correlated with increased FA in left precentral gyrus and posterior brain regions (Cheung et al., 2009), with reduced FA in the right anterior cingulate cortex (Thakkar et al., 2008) and with the number of tracts in the forceps minor (Thomas et al., 2011). More severe social-communicative deficits have been correlated with reduced FA in WM of fronto-striatal regions, temporal regions, the posterior part of the corpus callosum (Cheung et al., 2009), left and right uncinate fasciculus, left superior longitudinal fasciculus, left and right fornix (Poustka et al., 2011), the dorsolateral prefrontal cortex (Noriuchi et al., 2010), right anterior thalamic radiation and right uncinate fasciculus (Cheon et al., 2011) and left cerebellar peduncle (Catani et al., 2008) and with increased fiber length and density in the corpus callosum (Kumar et al., 2010). Alexander et al. found a relationship between Autism Spectrum Disorder (ASD) symptom severity (Social Responsiveness Scale) and WM measures (although it was only seen across both groups combined and not just within the ASD group; Alexander et al., 2007). Some studies however have found no correlation between ASD symptomatology and WM measures (Sundaram et al., 2008; Barnea-Goraly et al., 2010; Shukla et al., 2010; Hong et al., 2011; Jou et al., 2011). Reduced microstructural organization of WM in autism has also been correlated with lower performance IQ scores (Alexander et al., 2007) and with increased response times in a pictorial problem-solving task (Sahyoun et al., 2010).

A number of studies have also investigated the behavioral effects of functional connectivity abnormalities in ASD and have reported correlations between altered functional connectivity and core symptoms of autism. Reduced fronto-posterior functional connectivity was found to correlate with increased severity of autism (Just et al., 2007). Poorer social functioning in individuals with ASD has been associated with reduced functional connectivity between the superior frontal gyrus and posterior cingulate (Monk et al., 2009; Weng et al., 2010) and communication deficits have been associated with increased functional connectivity between regions of the default mode network during the resting state (Weng et al., 2010). Increased severity of repetitive behaviors in autism has been associated with reduced functional connectivity between frontal structures and the posterior cingulate (Weng et al., 2010), and also with increased functional connectivity between the posterior cingulate and parahippocampal gyrus (Monk et al., 2009) and between the anterior cingulate and frontal eye fields (Agam et al., 2010). In summary, results from both anatomical and functional studies suggest that disrupted neural connectivity in autism may impact negatively on core features of the condition.

### Evidence for a relationship between disrupted brain white matter structure and functional connectivity abnormalities in ASD

The direct impact of WM abnormalities on functional connectivity in ASD has been less well-studied. This is surprising given the extensive literature that has documented abnormal functional connectivity in the disorder, and the lack of knowledge about the pathophysiology of this abnormal functional connectivity. A small number of studies have used a measure of the size of the corpus callosum as an index of “anatomical connectivity” and demonstrated a relationship between reduced size of the corpus callosum and reduced functional connectivity during a number of neuropsychological paradigms, including the Tower of London task (Just et al., 2007), a sentence comprehension and visual imagery task (Kana et al., 2006), the Embedded Figures task (Damarla et al., 2010), a narrative comprehension task (Mason et al., 2008), and whilst participants were at rest (Cherkassky et al., 2006). No previous studies in autism have attempted to identify whether there are abnormal WM connections that directly link brain regions showing abnormal functional connectivity, nor have any studies examined the relationship between alterations in structural connectivity and functional connectivity.

### Evidence for a relationship between brain white matter structure and functional connectivity in neurotypical populations

In neurotypical populations, a number of multimodal (functional MRI and diffusion MRI) imaging studies have integrated structural and functional connectivity analyses in the same study population and have shown that there is evidence of substantial correspondence between structural and functional connectivity.

Studies that have combined analyses of structural and functional connectivity during the resting state have revealed a structural basis for resting state functional connectivity. Regions of the default mode network are linked by WM tracts (Greicius et al., 2009), the level of WM organization in the cingulum is correlated with resting state functional connectivity between midline brain regions (van den Heuvel et al., 2008) and maps of resting state functional connectivity in adult macaque monkeys were shown to be markedly similar to maps of structural connectivity obtained from tracer studies (Vincent et al., 2007; Margulies et al., 2009).

These combined structural/resting state studies have also provided evidence for a relationship (albeit complex) between anatomical and functional connectivity. Studies that have generated whole brain maps of functional and anatomical connectivity on the same cohort of participants have demonstrated that structural connection patterns and functional interactions between regions of the cortex are significantly correlated (Hagmann et al., 2008; Skudlarski et al., 2008; Honey et al., 2009; Hermundstad et al., 2013). This relationship between structural and functional connectivity is not a simple one however; strong functional connections often exist between regions with no direct anatomical connection (Honey et al., 2009). Such functional connections may arise from indirect WM connections, or from the two regions receiving common input from a third region (Behrens and Sporns, 2012).

There is also some evidence supporting a direct relationship between anatomical and functional connectivity at the level of individual pathways. The strength of functional connectivity within the default mode network was positively correlated with the level of WM organization in the cingulum, as estimated by FA (van den Heuvel et al., 2008). Consistent with the hypothesis that functional connectivity is directly related to anatomical connectivity, a study of three patients with callosal agenesis revealed reduced interhemispheric functional connectivity in the motor and auditory cortices (Quigley et al., 2003). Complete section of the corpus callosum in a young boy with intractable epilepsy resulted in a striking loss of interhemispheric resting state functional connectivity, with preservation of intrahemispheric functional connectivity (Johnston et al., 2008). In patients with multiple sclerosis, disease-related reduction of functional connectivity between left and right primary sensorimotor cortices was associated with increased radial diffusivity in the WM tracts connecting these regions, again indicating a relationship between reduced anatomical connectivity and reduced functional connectivity (Lowe et al., 2008).

To date, studies investigating a link between structural and functional connectivity in neurotypical populations have indicated that functional connectivity has a structural basis, and that there is evidence of substantial correspondence between the strength of structural and functional connectivity.

### Functional connectivity during visuospatial processing in ASD

Atypical visuospatial processing is common in autism spectrum disorders. In brief, enhanced visuospatial processing in ASD has been described in behavioral studies during a variety of cognitive tasks (see McGrath et al., 2012, for review). A number of neuroimaging studies have revealed that brain activity and connectivity differs markedly between ASD and control groups during visuospatial processing (Lee et al., 2007; Manjaly et al., 2007; Damarla et al., 2010). Recent work from our group used functional connectivity MRI (fcMRI) to investigate the neural correlates of visuospatial processing during a mental rotation task, whereby two rotated stimuli were judged to be the same (“Same” trials) or mirror-imaged (“Mirror” trials; McGrath et al., 2012). Results of this study indicated that there was a relative advantage of mental rotation in the ASD group. The ASD group performed Same and Mirror trials at similar speeds, but the control group slowed significantly on Mirror trials relative to Same trials. Functional connectivity analysis revealed marked abnormalities in the ASD group that were characterized by long-range fronto-posterior underconnectivity and short-range intra-occipital overconnectivity. This study concluded that atypical visuospatial processing in ASD appears to be associated with both quantitative and qualitative differences in functional connectivity, which may result in a combination of enhanced low-level visual perceptual processing and a reduction of higher-level cortical control. A further study from our group investigated the structural properties of major WM tracts that are thought to play an important role in visuospatial processing (McGrath et al., 2013) This research demonstrated that there were significant alterations in the microstructural organization of WM in the right inferior fronto-occipital fasciculus (IFOF) in ASD. This alteration was associated with poorer visuospatial processing performance in the ASD group. This study provided an insight into structural brain abnormalities that may influence atypical visuospatial processing in autism, however it did not provide any information on how WM abnormalities may impact on functional connectivity in the disorder.

### Aims and hypotheses

In ASD, there is strong evidence for disrupted functional and anatomical connectivity but no previous studies have attempted to integrate these types of connectivity analyses. The integration of functional and structural connectivity analysis in the same study population allows for the investigation of inter-participant variability in structural connectivity and provides an opportunity to relate this variability to differences in individual functional connectivity and behavior (Hagmann et al., 2008). As discussed above, such multimodal connectivity studies in neurotypical populations have revealed strong relationships between WM organization, functional connectivity and behavior. Thus, the aims of this study were two-fold; first to investigate the structural integrity of WM that directly connected brain regions showing abnormal functional connectivity in ASD, and second to investigate relationships between brain WM structure, functional connectivity and behavior. To do this, brain regions from a previously reported functional connectivity analysis (McGrath et al., 2012) were used as regions of interest (ROIs) for diffusion tractography in order to isolate WM tracts that directly linked the two regions. Microstructural organization of these WM tracts was assessed and correlated with both functional connectivity and behavioral measures to provide a comprehensive examination of the relationships between brain structural connectivity, functional connectivity and behavior in ASD. It was hypothesized that there would be WM tracts linking some, but not all, pairs of brain regions that showed abnormal functional connectivity. This hypothesis was based on the knowledge that functional connectivity between regions does not always require a direct WM connection, but can be mediated by indirect connections or input from unrelated regions (Behrens and Sporns, 2012). It was also hypothesized that WM structure would be abnormal in tracts directly connecting the functionally defined ROIs and that there would be correlation between microstructural organization of WM, functional connectivity and behavior. It is difficult to make specific predictions about the correlations in this study because there is such a limited literature that has investigated relationships between structural connectivity, functional connectivity and behavior. Nevertheless, it was theorized that if ASD and control groups do use qualitatively and quantitatively neural networks for successful visuospatial processing as hypothesized in McGrath et al. (2012), there should be differential relationships between WM organization, functional connectivity and response time data in the ASD and control groups.

## Materials and methods

### Participants

Twenty-two right-handed male individuals with ASD and 22 right-handed age- and IQ-matched male neurotypical controls were included in the analysis (see Table 1). Participants with ASD were recruited from an existing autism genetics sample at the Department of Psychiatry, Trinity College Dublin, and through additional recruitment from local schools and child and adolescent mental health services. The diagnosis of autism was established using two structured research diagnostic tools; the Autism Diagnostic Interview-Revised [ADI-R, (Lord et al., 1994)] and the Autism Diagnostic Observation Schedule-Generic [ADOS-G, (Lord et al., 2000)]. Administrators of the ADI-R and ADOS-G were trained to reliability and maintained reliability. Community-recruited control participants were selected to match participants with autism on age, handedness, gender, race and IQ (full-scale IQ was estimated based on four sub-scales of the WISC/WAIS [Wechsler Intelligence Scale for Children (WISC-III and IV UK)], (Wechsler, 2004) and Wechsler Adult Intelligence Scale (WAIS-III), (Wechsler, 1997)]. Verbal IQ and performance IQ were estimated from the verbal and performance subtests of the WISC-III and WAIS-III data. Exclusion criteria included known causes for autism, e.g., tuberous sclerosis/fragile-X syndrome, current/past neurological or psychiatric conditions, serious head injuries, MR contraindications, below-average intelligence (full-scale IQ <80) and current use of psychoactive medication. Additional exclusion criteria for controls included history of developmental delay or first-degree relatives with ASD. The study was approved by the Irish Health Services Executive Linn Dara-Beechpark Research Ethics committee and by the School of Psychology Ethics Committee, Trinity College Dublin. Written informed consent was obtained from parents (where appropriate) and participants prior to scanning. Demographics of participants are detailed in Table 1.

**Table 1:** Demographics of study participants ^*^Full scale IQ was estimated based on four sub-scales of the WISC/WAIS [Wechsler Intelligence Scale for Children (WISC-III or IV UK), (Wechsler, 2004) and Wechsler Adult Intelligence Scale (WAIS-III), (Wechsler, 1997)].

	**Control**	**ASD**	***p*-value**
Number	22	22	
Gender	Male (22)	Male (22)	
Age mean (SD, Range)	17.51 (2.76, 13.6–21.3)	17.56 (2.91, 13.0–21.8)	0.76
Full scale IQ^*^ Mean (SD, Range)	110.50 (16.97, 84–147)	105.95 (13.46, 84–127)	0.25
Verbal IQ^∧^ Mean (SD, Range)	117.60 (12.69, 93–134)	105.60 (18.48, 79–134)	0.11
Performance IQ~ Mean (SD, Range)	120.80 (26.39, 91–155)	124.35 (17.46, 99–155)	0.73
Handedness	Right (22)	Right (22)	
Medication	None	None	
Ethnicity	Irish (22)	Irish (22)	

∧ Verbal IQ was estimated using the Information and Vocabulary subtests of the WISC III for n = 10 participants with ASD and n = 10 matched controls, using Sattler's method (Sattler, 1992). It was not possible to produce a Verbal IQ for n = 12 participants with ASD and n = 12 controls, as the WISC IV was used to estimate full scale IQ for these participants. ~Performance IQ was estimated using the Picture Completion and Block Design subtests of the WISC III for n = 10 participants with ASD and n = 10 for controls, using Sattler's method (Sattler, 1992). It was not possible to produce a Performance IQ for n = 12 participants with ASD and n = 12 controls, as the WISC-IV was used to estimate full scale IQ for these participants. Subtests of the WISC-IV that were used to calculate full scale IQ included Similarities, Block Design, Digit Span, and Coding. Subtests of the WAIS that were used to calculate full scale IQ included Similarities, Block Design, Digit Span and Matrix Reasoning.

### Overview of methods

To investigate the links between brain structural connectivity and functional connectivity, diffusion MRI data, and previously analysed functional connectivity data (see McGrath et al., 2012) from the same participants were combined. Three questions were posed:

1. Were there WM tracts directly linking pairs of brain regions that showed abnormal functional connectivity in the ASD group?
2. If there were WM tracts that directly linked pairs of brain regions showing abnormal functional connectivity, were there structural abnormalities of this WM in the ASD group?
3. Was there evidence for relationships between structural connectivity, functional connectivity and behavior?

To answer question 1, ROIs were defined from the results of a previously reported functional connectivity analysis, in which significant abnormalities of functional connectivity were reported in the ASD group (McGrath et al., 2012). These functionally defined ROIs were then used in diffusion tractography analysis to determine whether WM tracts linked brain regions that were abnormally functionally connected in the ASD group. In the methods sections below, there is a brief overview of the previously reported functional connectivity analysis (section Review of Functional Connectivity Analysis), a description of the diffusion MRI acquisition and pre-processing (section Diffusion MRI Acquisition/Preprocessing), a description of how functionally defined ROIs for tractography were selected (section Selection of Functionally Defined Seed Regions for Diffusion Tractography) and prepared for diffusion tractography (section Preparation of Functionally Defined ROIs for Tractography), and a description of the diffusion tractography protocol (section Diffusion Tractography Protocol).

To answer question 2, diffusion measures were extracted from the isolated tracts and compared between groups. Methods for this analysis are outlined in sections Dependent Measures and Between-Group Differences in White Matter Structure.

To answer question 3, a series of exploratory correlation analyses were performed to investigate the relationships between structural connectivity, functional connectivity and behavior (visuospatial processing speed). Methods for the correlation analyses are outlined in sections Correlation Analyses and Measures of White Matter Structure, Functional Connectivity and Behavior Included in the Correlation Analyses.

### Review of functional connectivity analysis

fcMRI data was available on all participants who completed the diffusion MRI scan. In a previous study (McGrath et al., 2012), *psychophysiological interaction (PPI) functional connectivity analysis* (Friston et al., 1997) was used to examine functional connectivity between six seed ROIs and the rest of the brain during performance of a mental rotation task. Please see McGrath et al. (2012) for full details of the mental rotation task, functional MRI acquisition, and PPI functional connectivity analysis. A brief summary of the functional connectivity analysis performed in McGrath et al. (2012) follows. PPI analyses were performed separately for each ROI for the Same and Mirror trials for ASD and control groups. For each PPI analysis, a multiple regression analysis was carried out for each subject. This analysis comprised seven task-related regressors (one for each experimental condition in the mental rotation task) and the motion-corrected time-series regressors to accommodate nuisance variance (for fMRI analysis). In addition, there were two other regressors. The first regressor, the physiological variable, was the detrended subject-specific time course of activity in the ROI (averaged across all voxels in the 8 mm sphere). The second regressor—the PPI term—was created by calculating the product of the detrended activation time-course from the seed region and the task regressor. The parameter estimate for the interaction term was converted to a Z-score through Fisher transformation for each subject. To investigate between-group differences in functional connectivity, Z-scores from each PPI analysis were entered into a Two-Way repeated-measures ANOVA [Group (ASD/Control)× Trial-type (Same/Mirror)]. The dependent variables that result from this PPI analysis are negative and positive connectivity. Negative connectivity indicates that the influence the task has on activity in the seed region produces a correlated opposite effect on the correlated region, which is consistent with (but not proof of) one region suppressing the other. In contrast positive connectivity between a pair of brain regions indicates that as activity in one brain region increases, there is a correlated increase in activity in the other region.

This previous study identified between-group differences in functional connectivity between all seed ROIs and numerous brain regions. These findings are summarized in Table 2, which is a modified version of Table 3 from McGrath et al. (2012).

**Table 2:**
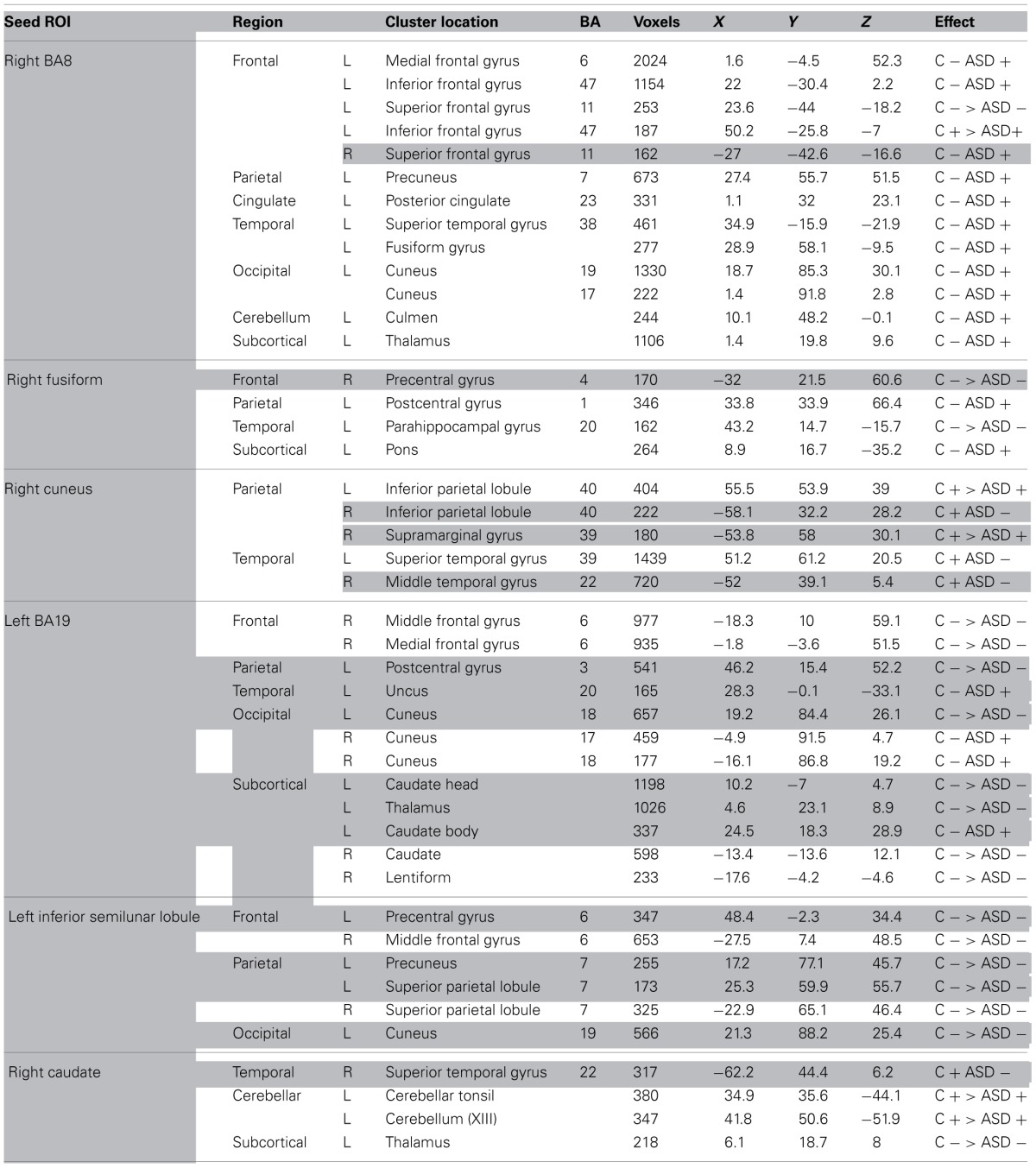
**A modified version of Table 3 in McGrath et al. (2012), in which the results of between-group differences in functional connectivity are summarized**.

Gray shading indicates ipsilateral brain region pairs that were used as regions of interest for CSD based tractography in the current study. [Direction of between-group difference denoted with arrows (–, Negative functional connectivity, +, Positive functional connectivity), >, greater than (note that when both groups show negative connectivity the > means a larger negative value), C, Control group, ASD, Autism Spectrum Disorder group].

### Diffusion MRI acquisition/preprocessing

Whole-brain high angular resolution diffusion imaging (HARDI) data were acquired on a Philips Intera Achieva 3.0 Tesla MR system (Best, Netherlands) equipped with an eight-channel head coil. A parallel Sensitivity Encoding (SENSE) approach (Pruessmann et al., 1999) with a reduction factor of 2 was utilized for all diffusion weighted image (DWI) acquisitions. Single shot spin echo-planar imaging was used to acquire diffusion weighted data using the following parameters (Jones and Leemans, 2011): echo time (TE) 79 ms, repetition time (TR) 20,122 ms, field of view 248 × 248 mm^2^, matrix 128 × 128, isotropic voxel resolution 2 × 2 × 2 mm^3^, 65 slices with 2 mm thickness with no gap between slices. Diffusion gradients were applied in 61 isotropically distributed orientations with *b* = 1500 s/mm^2^ and also four images with *b* = 0 s/mm^2^ were acquired. Total DWI scan time was 24.3 min.

Pre-processing and tractography analyses were performed with the diffusion MR toolbox ExploreDTI (http://www.ExploreDTI.com; Leemans et al., 2009). Each DW-MRI dataset was corrected for eddy current induced geometric distortions and subject motion by realigning all DWIs to the *b* = 0 images using Elastix (Klein et al., 2010), with an affine co-registration technique (with 12 degrees of freedom) and mutual information as the cost function (Pluim et al., 2003). In this procedure, the required reorientation of the B-matrix was performed (Leemans and Jones, 2009) and the tensor model was fitted to the data using the RESTORE approach (Chang et al., 2005), which uses a process of iteratively reweighted least-square regression for outlier identification and subsequent removal, thus minimizing estimation errors originating from gross signal artifacts (e.g., cardiac pulsation and subject motion).

### Selection of functionally defined seed regions for diffusion tractography

Results of the functional connectivity analysis, discussed in detail in McGrath et al. (2012), revealed that there were significant group differences in functional connectivity between a large number of brain regions (see Table 2). In the current study, pairs of brain regions showing abnormal functional connectivity in ASD were used as ROIs for diffusion tractography analysis. To minimize the number of tractography analyses, only ipsilateral pairs of brain regions that showed abnormal functional connectivity were selected for analysis (i.e., the pair of brain regions had to be either in right or left hemisphere). The analysis was limited to ipsilateral pairs as it was thought less likely that there would be direct long-range WM connections between left and right hemispheres. For example, unless the two regions showing abnormal connectivity both were in the region of the corpus callosum, it was unlikely that there would be one direct WM tract linking them. In total there were 16 ipsilateral pairs of brain regions that showed abnormal functional connectivity in ASD in this previous study. These are shown shaded in Table 2, which is a modified version of Table 3 in McGrath et al. (2012), in which the results of between-group differences in functional connectivity are summarized.

### Preparation of functionally defined ROIs for tractography

To prepare the 16 pairs of ROIs for tractography, each cluster of interest was isolated from the fcMRI analysis and was projected back from standard MNI space into the space of the original subjects' diffusion data (native space) using the FSL TBSS Deproject tool (http://www.fmrib.ox.ac.uk/fsl/tbss/index.html). These ROIs were subsequently used to select the fiber trajectories that were computed with the constrained spherical deconvolution (CSD) based tractography approach (discussed in section Diffusion Tractography Protocol below).

Three of these ROIs (namely, the right superior frontal gyrus, right superior temporal gyrus, and left inferior semilunar lobule) failed to deproject successfully into native space. This failure to deproject occurred because the fcMRI clusters fell solely within the external extremity of the gray matter cortex and did not extend into subcortical WM regions. These subcortical regions are used by (and required by) the TBSS analysis during construction of the FA skeleton that is needed for transformation between native and standard space. Therefore, after deprojection, there were 10 pairs of brain regions in native diffusion space that were used as ROIs for CSD-based tractography. The location of these 10 pairs of brain regions are reported in Table 3.

**Table 3:** **Summary of results from functional connectivity and tractography analysis**.

**ROI 1**	***X***	***Y***	***Z***	**ROI 2**	***X***	***Y***	***Z***	**Mean functional connectivity**	**Tractography result**
Right Fusiform	−55	51	−19	Right precentral gyrus	−32	22	61	C −0.046 ASD −0.013	No direct tracts
Right Cuneus	−11	75	6	Right inferior parietal lobule	−58	32	28	C +0.029 ASD −0.013	No direct tracts
				Right supramarginal gyrus	−54	58	30	C +0.046 ASD +0.004	No direct tracts
				Right middle temporal gyrus	−52	39	5	C +0.017 ASD −0.018	No direct tracts
Left BA19	42	73	−2	Left postcentral gyrus	46	15	52	C −0.059 ASD −0.010	No direct tracts
				Left caudate head	10	−7	5	C −0.050 ASD −0.011	Tracts in 9 controls, 11 ASD
				Left thalamus	5	23	9	C −0.051 ASD −0.003	Tracts in 12 controls, 13 ASD
				Left caudate body	25	18	29	C −0.004 ASD +0.027	Tracts in 22 controls, 22 ASD
				Left cuneus	19	84	26	C − 0.066 ASD −0.010	Tracts in 22 controls, 22 ASD
				Left Uncus	28	0	−33	C −0.020 ASD +0.005	Tracts in 5 controls, 6 ASD

ROI_1 and ROI_2 refer to the regions used in tractography analysis. [Direction of between-group difference for functional connectivity is denoted with arrows (−, Negative functional connectivity, +, Positive functional connectivity), C, Control group, ASD, Autism Spectrum Disorder group].

### Diffusion tractography protocol

CSD based tractography was used in this study. Typically a model of diffusion tensor tractography has been used in ASD research, however in recent years it has become evident that there are significant limitations associated with this method, in particular in voxels containing more than one coherently oriented fiber population (e.g., in “crossing fibers” configurations; Wedeen et al., 2000; Alexander et al., 2002; Frank, 2002; Wedeen et al., 2008; Tournier et al., 2011; Jeurissen et al., 2012). The CSD method allows reliable estimation of one or more fiber orientations in the presence of intra-voxel orientational heterogeneity (Tournier et al., 2004, 2007, 2008), it overcomes partial volume effects associated with diffusion tensor imaging (Vos et al., 2011, 2012), permits fiber-tracking through regions of crossing fibers (Tournier et al., 2008), and has recently shown promising results in other clinical applications (Metzler-Baddeley et al., 2012; Reijmer et al., 2012, 2013).

The WM tracts between each pair of ROIs were reconstructed using CSD based tractography (Jeurissen et al., 2011). This tractography procedure consisted of the following steps: (i) CSD, using a spherical harmonics model with maximum harmonic degree *L* = 8 was used to extract the fiber orientation distribution (FOD) from the diffusion weighted signal in each voxel (Tournier et al., 2007), (ii) Seed points were defined on a uniform 2 × 2 × 2 mm^3^ grid to cover the entire brain; (iii) For each step during tract propagation, the FOD peak direction that was closest to the previous stepping direction was extracted; (iv) The trajectory was advanced with a fixed step size (1 mm) along the peak direction obtained with step (iii). Tracking ended when the FOD peak magnitude was beneath a fixed threshold (i.e., 0.1), or when a maximum angle (30°) was exceeded. Subsequently, from this whole-brain tractography result, WM tracts that ran directly between each pair of ROIs were identified by coding the two regions as “AND” regions (i.e., only WM tracts that passed through ROI 1 and ROI 2 were isolated).

Preliminary tractography analysis was carried out using the exact clusters from functional connectivity analysis as outlined in Table 2, however this analysis indicated that there were no (or very few) WM tracts between the pairs of brain regions. One possible reason for this is that these are functionally defined ROIs, which are essentially generated by BOLD signal fluctuation in gray matter, and therefore they may not have projected far enough into the adjacent WM. A pragmatic approach was thus adopted using larger ROIs in the analysis to increase projection into WM. These larger ROIs were created in standard space by generating a sphere 8 × 8 mm for each cluster with its center-point the center of mass of the original fcMRI derived cluster. This size of sphere was chosen as it corresponded with the size of the seed spheres used in the corresponding functional connectivity analysis (McGrath et al., 2012). These spheres were then back projected from standard MNI space to each participants' native diffusion space and used as the ROIs for CSD based tractography as outlined above. All tractography analyses were performed in native diffusion space.

### Dependent measures

For each tract in each participant, microstructural measures of FA and the Westin measures of linear diffusion coefficient (CL) and planar diffusion coefficient (CP; Westin et al., 2002) were computed from the tracts. The mean values for FA, CL, and CP were extracted from all tracts using Explore DTI software (Leemans et al., 2009). FA was the primary measure of interest as it is the most widely used measure in the literature, and shows high sensitivity. In regions of complex fiber architecture however, tensor derived measures such as FA are unreliable (Jones and Cercignani, 2010) and the interpretation of these measures can be ambiguous (Jeurissen et al., 2012) and see McGrath et al. (2013) for discussion. In order to reduce the ambiguity about the biological interpretation of FA changes, alternative measures of diffusion anisotropy are often measured in conjunction with FA. One example of such alternative tensor-based metrics are the Westin measures of CL and CP. Although these measures are indeed still based on the eigenvalues, they can describe the geometrical shape of the diffusion tensor and, therefore, can provide a more meaningful interpretation of microstructural changes that are occurring in the ASD group compared to the FA (Westin et al., 2002; Reijmer et al., 2013). A high value of CL implies that there is only one dominant fiber orientation within a voxel (Vos et al., 2012) and a high value of CP indicates the presence of crossing fiber configurations (Vos et al., 2012).

### Statistical analysis

#### Between-group differences in white matter structure

Statistical comparisons of the data were performed using PASW (SPSS) software version 18 (SPSS Inc., Chicago, IL). For all analyses the level of statistical significance was defined as *p* < 0.05 (two-tailed) and Bonferroni corrections were used for within-test comparisons. To investigate whether there were between-group differences in the WM of tracts that directly connected a pair of brain regions, univariate ANOVA with Group (ASD/Control) as the between-subjects factor was performed for the dependent measures FA, CL, and CP in each separate set of these WM tracts.

#### Correlation analyses

To explore how brain WM structure, functional connectivity and behavior are related, a number of exploratory correlation analyses (using bivariate Pearson correlation analysis) were performed to investigate the relationships between (1) WM structure and functional connectivity, (2) WM structure and behavior, (3) functional connectivity and behavior. Given the extremely limited data in current literature, in both autism and healthy populations, on relationships between brain structural connectivity, functional connectivity, and behavior, it was felt important that all these measures were included to comprehensively explore the possible associations. Pearson correlation analysis was used as behavioral response times, fcMRI and DTI data in this study are normally distributed, as indicated by *p*-values of >0.05 following Kolmgorov–Smirnov and Shapiro–Wilk tests of normality. The correlation analyses were exploratory in nature, and correction for multiple comparisons was not performed.

***Measures of white matter structure, functional connectivity and behavior included in the correlation analyses.*** For WM structure, FA was included in the correlation analysis. This measure of WM structure is the most widely used measure in the literature, and shows high sensitivity.

Four measures of functional connectivity were included in the correlation analyses; negative functional connectivity on Same trials, negative functional connectivity on Mirror trials, positive functional connectivity on Same trials and positive functional connectivity on Mirror trials. The distinction between Same and Mirror trials was included in the correlations as behavioral and functional connectivity analyses both demonstrated an interesting dissociation between ASD and control groups on Same vs. Mirror trials (McGrath et al., 2012). These findings may indicate that visuospatial processing in ASD is achieved using qualitatively (and quantitatively) different neural networks. A primary aim of the current study was to increase our understanding of the structural correlates of atypical visuospatial processing in ASD. We hypothesized that correlation analyses would demonstrate differential relationships between ASD and control groups on Same and Mirror trials. A distinction was also made between negative and positive functional connectivity. Unfortunately, there is very limited literature investigating the relationships between structural and functional connectivity in neurotypical populations, therefore it is not possible to make specific predictions about how structural connectivity may relate differentially to these types of functional connectivity. Nevertheless, there is an important difference between these measures in terms of functional interactions between brain regions (see McGrath et al., 2012, for discussion). While it seems plausible that the level of WM organization should be correlated with the overall strength of functional connectivity, it was felt that it would not be appropriate to combine negative and positive functional connectivity into a composite measure as doing so might obscure important relationships between WM organization and functional connectivity. Functional connectivity values were individual z-scores extracted from the PPI Main effect of Group results. These were extracted for each participant for each pair of brain regions in which there were WM connections.

The behavioral data used in the correlation analyses provided a measure of visuospatial processing speed. This was calculated using mean response times (MRTs) for the Same and Mirror trials during a mental rotation task (McGrath et al., 2012).

A set of correlation analyses was performed for every set of brain regions that had direct WM connections. The groups (ASD and controls) were analysed separately for the correlation analyses.

## Results

### Overview of results

#### Section 3.1

Results relate to Question 1 outlined in Methods section Overview of Methods—are there WM tracts between all pairs of ROIs that are functionally connected? This section includes a summary of the between-group differences in functional connectivity, which are reported in McGrath et al. (2012).

#### Section 3.2

Results relate to Question 2 outlined in the Methods section Overview of Methods—if there were WM tracts that directly linked pairs of brain regions showing abnormal functional connectivity, were there structural abnormalities of this WM in the ASD group? This section outlines the between-group comparisons of diffusion measures extracted from the isolated WM tracts.

#### Section 3.3

Results relate to Question 3 outlined in the Methods section Overview of Methods—was there evidence for relationships between structural connectivity, functional connectivity and behavior? This section outlines the results of the correlation analyses.

### Are there white matter tracts between all pairs of ROIs that are functionally connected?

**Figure 1:**
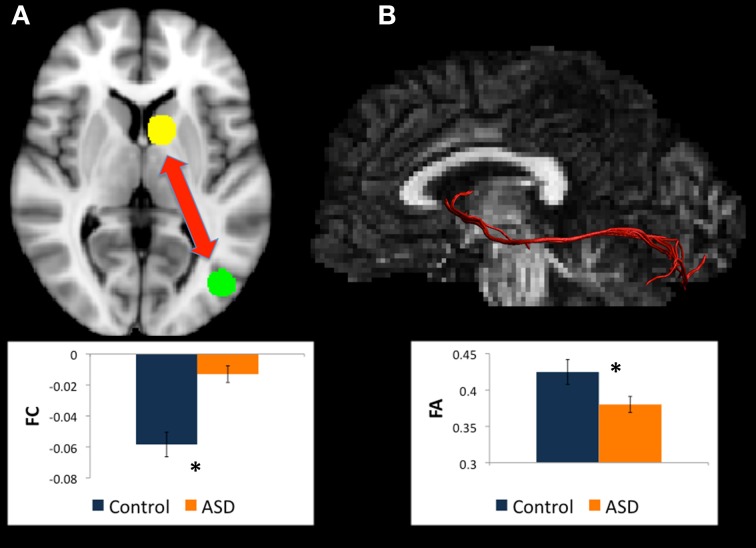
**(A)** Regions for tractography in left BA19 (green sphere) and left caudate head (yellow sphere). The ASD group showed weaker functional connectivity relative to controls between these regions during a mental rotation task. The bar graph shows the strength of functional connectivity (FC) in the Control group (blue) and ASD group (orange) between these seed regions. **(B)** Example of white matter tracts that directly connect the left BA19 and left caudate head regions in one participant. The bar graph shows Fractional Anisotropy (FA) in the Control group (blue) and ASD group (orange) in these white matter tracts. ^*^indicates statistical significance with *p* < 0.05.

**Figure 2:**
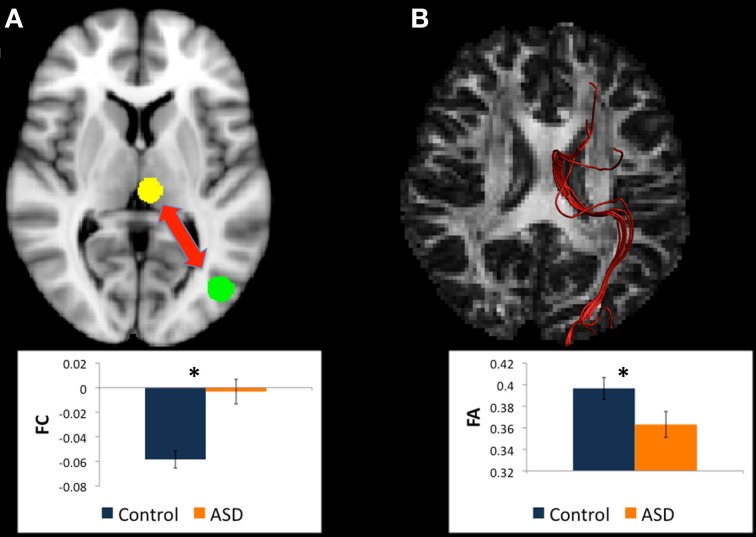
**(A)** Regions for tractography in left BA19 (green sphere) and left thalamus (yellow sphere). The ASD group showed much weaker functional connectivity relative to controls between these regions during a mental rotation task. The bar graph shows the strength of functional connectivity (FC) in the Control group (blue) and ASD group (orange) between these regions. **(B)** Example of white matter tracts that directly connect the left BA19 and left thalamus regions in one participant. The bar graph shows Fractional Anisotropy (FA) in the Control group (blue) and ASD group (orange) in these white matter tracts. ^*^indicates statistical significance with *p* < 0.05.

**Figure 3:**
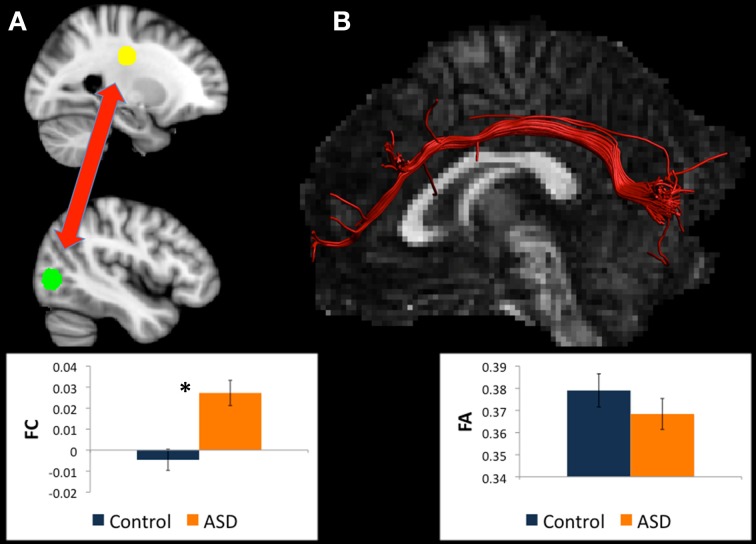
**(A)** Regions for tractography in left BA19 (green sphere) and left caudate body (yellow sphere). The ASD group showed increased functional connectivity relative to controls between these regions during a mental rotation task. The bar graph shows the strength of functional connectivity (FC) in the Control group (blue) and ASD group (orange) between these regions. **(B)** Example of white matter tracts that directly connect the left BA19 and left caudate body regions in one participant. The bar graph shows Fractional Anisotropy (FA) in the Control group (blue) and ASD group (orange) in these white matter tracts. ^*^indicates statistical significance with *p* < 0.05.

**Figure 4:**
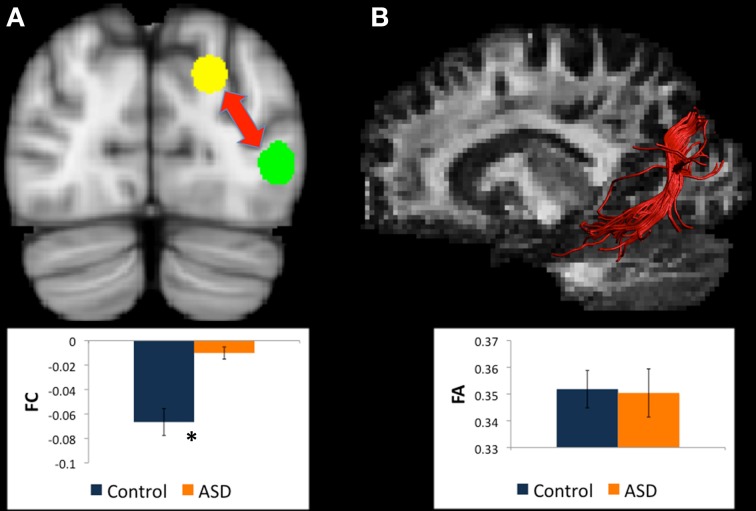
**(A)** Regions for tractography in left BA19 (green sphere) and left cuneus (yellow sphere). The ASD group showed weaker functional connectivity relative to controls between these regions during a mental rotation task. The bar graph shows the strength of functional connectivity (FC) in the Control group (blue) and ASD group (orange) between these regions. **(B)** Example of white matter tracts that directly connect the left BA19 and left cuneus regions in one participant. The bar graph shows Fractional Anisotropy (FA) in the Control group (blue) and ASD group (orange) in these white matter tracts. ^*^indicates statistical significance with *p* < 0.05.

**Figure 5:**
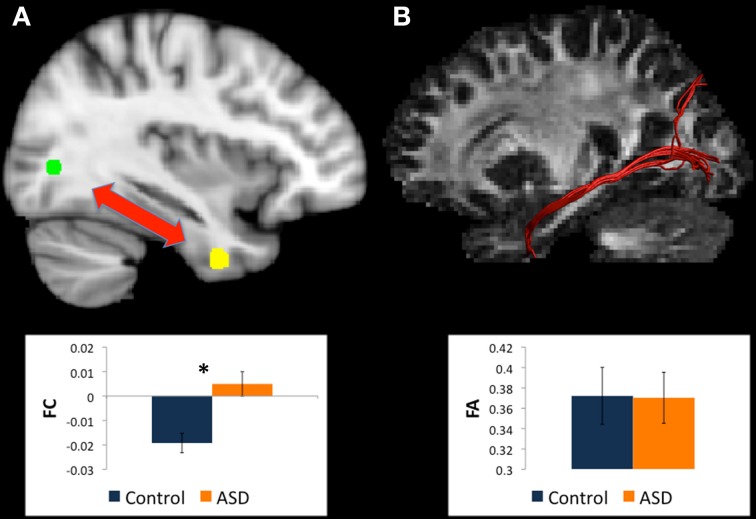
**(A)** Regions for tractography in left BA19 (green sphere) and left uncus (yellow sphere). The ASD group showed weaker functional connectivity relative to controls between these regions during a mental rotation task. The bar graph shows the strength of functional connectivity (FC) in the Control group (blue) and ASD group (orange) between these regions. **(B)** Example of white matter tracts that run through the left BA19 and left uncus regions in one participant. The bar graph shows Fractional Anisotropy (FA) in the Control group (blue) and ASD group (orange) in these white matter tracts. ^*^indicates statistical significance with *p* < 0.05.

Table 3 summarizes the results of the tractography analyses between the 10 pairs of ROIs that showed abnormal functional connectivity. In summary, there were WM tracts directly connecting the left BA19 ROI to five other ROIs including the left uncus, left cuneus, left caudate head, left caudate body, and left thalamus (see Figures 1–5). There were no WM tracts in any participants between the other five pairs of seed regions. Consequently, the subsequent analyses of diffusion measures and the correlation analyses are restricted to the five pairs of ROIs with direct WM tracts.

### If there were white matter tracts that directly linked pairs of brain regions showing abnormal functional connectivity, were there structural abnormalities of this white matter in the ASD group?

#### Changes in white matter between left BA19 and left caudate head

There were WM tracts directly connecting the regions in left BA19 and left caudate head in 9 controls and 11 participants with ASD, and the WM tracts linking these regions formed part of the left IFOF (see Figure 1). Univariate ANOVA of the dependent measures from diffusion analysis revealed that there was significantly reduced FA (mean FA ASD 0.38, *SD* 0.04, controls 0.42, *SD* 0.05, *F* = 5.972, *p* = 0.027, η^2^_*p*_ = 0.272) and CL (mean CL ASD 0.35, *SD* 0.04, controls 0.40, *SD* 0.06, *F* = 5.074, *p* = 0.039, η^2^_*p*_ = 0.199) in the ASD group relative to controls (Table 4), indicating that WM microstructural organization was reduced in these tracts in the ASD group.

**Table 4:** **The mean, standard deviation and *p*-values for the three micro-structural dependent measures (FA, CP, CL) for white matter tracts connecting functionally defined regions in the ASD and control groups**.

**Tract**		**Left BA19 − Left uncus**		**Left BA19 − Left cuneus**		**Left BA19 − Left caudate head**		**Left BA19 − Left thalamus**		**Left BA19 − Left caudate body**	
					
		**ASD *n* = 6**		**ASD *n* = 22**		**ASD *n* = 9**		**ASD *n* = 12**		**ASD *n* = 22**	
		**Con *n* = 5**		**Con *n* = 22**		**Con *n* = 11**		**Con *n* = 13**		**Con *n* = 22**	
					
**Measure**	**Group**	**Mean (*SD*)**	***p***	**Mean (*SD*)**	***p***	**Mean (*SD*)**	***p***	**Mean (*SD*)**	***p***	**Mean (*SD*)**	***p***
FA	ASD	0.37 (0.06)	0.69	0.35 (0.04)	0.98	0.38^*^ (0.04)	0.02^*^	0.36^*^ (0.04)	0.05^*^	0.37 (0.03)	0.23
	Con	0.37 (0.07)		0.35 (0.03)		0.42^*^ (0.05)		0.39^*^ (0.04)		0.38 (0.03)	
CP	ASD	0.16 (0.03)	0.65	0.17 (0.03)	0.81	0.16 (0.02)	0.93	0.16 (0.04)	0.48	0.19 (0.03)	0.85
	Con	0.17 (0.04)		0.17 (0.02)		0.16 (0.01)		0.15 (0.02)		0.19 (0.02)	
CL	ASD	0.35 (0.05)	0.78	0.32 (0.05)	0.96	0.35^*^ (0.04)	0.04^*^	0.34^**^ (0.04)	0.01^**^	0.33 (0.03)	0.26
	Con	0.34 (0.08)		0.32 (0.04)		0.40^*^ (0.06)		0.38^**^ (0.03)		0.34 (0.03)	

* Indicates statistical significance p < 0.05;

** indicates statistical significance p < 0.01.

#### Changes in white matter between left BA19 and left thalamus

There were WM tracts directly connecting left BA19 and left thalamus in 12 controls and 13 participants with ASD, and this WM appeared to comprise part of the left IFOF (see Figure 2). Univariate ANOVA revealed significantly reduced FA (mean FA ASD 0.36, *SD* 0.04, controls 0.40, *SD* 0.04, *F* = 4.306, *p* < 0.050, η^2^_*p*_ = 0.170) and CL (mean CL ASD 0.34, *SD* 0.04 controls 0.38, *SD* 0.03, *F* = 8.085, *p* < 0.010, η^2^_*p*_ = 0.278) in the ASD group relative to controls in the WM directly connecting left BA19 and left thalamus regions.

#### Changes in white matter between left BA19 and left caudate body

There were WM tracts directly connecting left BA19 and left caudate body in 22 controls and 22 participants with ASD, and this WM appeared to be part of the superior longitudinal fasciculus (see Figure 3). There were no between-group differences in microstructural measures of the WM linking left BA19 and left caudate body.

#### Changes in white matter between left BA19 and left cuneus

There were WM tracts directly connecting the regions in left BA19 and left cuneus in 22 controls and 22 participants with ASD (see Table 3). This WM tract ran intra-occipitally in the left hemisphere (see Figure 4). There were no between-group differences in microstructural organization of this tract (see Table 4).

#### Changes in white matter between left BA19 and left uncus

There were WM tracts directly connecting the regions in left BA19 and the left uncus in only 5 controls and 6 participants with ASD (see Table 3). This WM appeared to be part of the left IFOF/left inferior longitudinal fasciculus (see Figure 5). There were no between-group differences in WM microstructure in this tract (see Table 4).

### Is there evidence for relationships between structural connectivity, functional connectivity and behavior?

#### Correlation analyses of behavioral measures, diffusion measure, and functional connectivity in left ba19/left caudate head region

**Figure 6:**
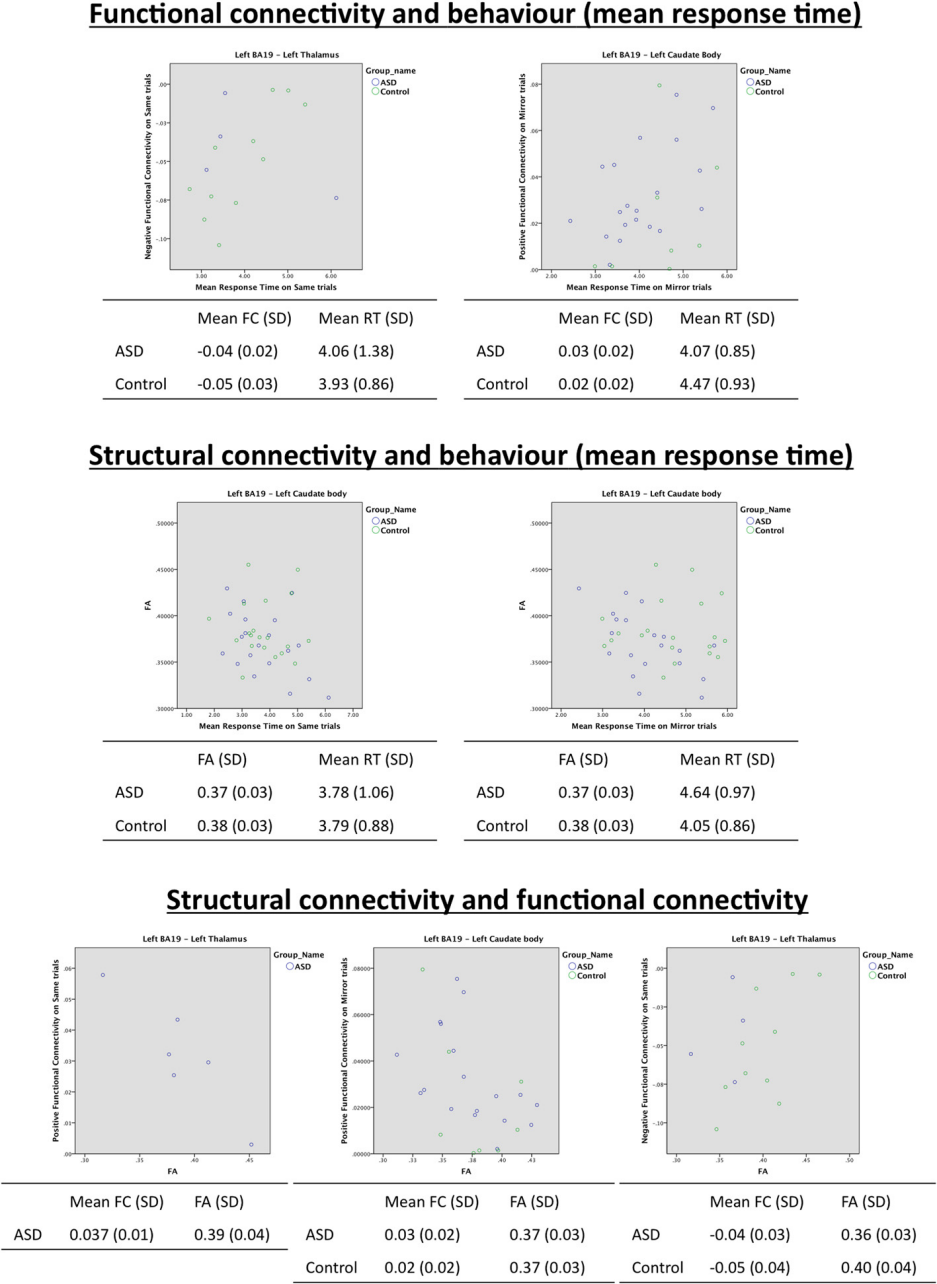
**Scatterplots of correlation analyses showing significant relationships between functional connectivity, structural connectivity and behavior**.

For both control and ASD groups, no significant correlations were found between the functional connectivity and mean RT measures (Table 5), FA and mean RTs (Table 6), or FA and functional connectivity measures (Table 7 and see Figure 6).

**Table 5:** **Results of correlation analysis between functional connectivity and mean response times during Same and Mirror trials of a mental rotation task (*p*: *p*-value, r: Pearson correlation co-efficient, FC_S − Negative functional connectivity on Same trials, FC_S + Positive functional connectivity on Same trials, FC_M − Negative functional connectivity on Mirror trials, FC_M + Positive functional connectivity on Mirror trials, MRT: mean response time, ^∧^1 Insufficient data for correlation as all functional connectivity values were negative between seed regions in left BA19 and left caudate head)**.

			**MRT_S *r* (*p*)**			**MRT_M *r* (*p*)**
Left BA19 − Left cuneus	FC_S −	ASD	0.18 (0.53	FC_M −	ASD	0.16 (0.55)
		Control	−0.17 (0.51)		Control	0.02 (0.93)
	FC_S +	ASD	−0.52 (0.29)	FC_M +	ASD	0.03 (0.97)
		Control	−0.08 (0.92)		Control	^∧^1
Left BA19 − Left caudate head	FC_S −	ASD	−0.22 (0.58)	FC_M −	ASD	0.03 (0.95)
		Control	0.63 (0.07)		Control	0.15 (0.69)
	FC_S +	ASD	^∧^1	FC_M +	ASD	^∧^1
		Control	^∧^1		Control	^∧^1
Left BA19 − Left thalamus	FC_S −	ASD	−0.63 (0.37)	FC_M −	ASD	0.17 (0.57)
		Control	0.80 (0.006)^**^		Control	0.18 (0.42)
	FC_S +	ASD	−0.35 (0.33)	FC_M +	ASD	−0.16 (0.77)
		Control	^∧^1		Control	^∧^1
Left BA19 − Left caudate body	FC_S −	ASD	−0.50 (0.31)	FC_M −	ASD	^∧^1
		Control	−0.03 (0.93)		Control	−0.18 (0.53)
	FC_S +	ASD	0.23 (0.36)	FC_M +	ASD	0.53 (0.02)^*^
		Control	0.53 (0.14)		Control	0.34 (0.41)

* Indicates statistical significance p < 0.05;

** indicates statistical significance p < 0.01.

**Table 6:** **Results of correlation analysis between mean response times during a mental rotation task and the micro-structural diffusion measure of FA extracted from the white matter tracts linking functionally defined regions**.

	**Left BA19 − Left cuneus**		**Left BA19 − Left caudate head**		**Left BA19 − Left thalamus**		**Left BA19 − Left caudate body**	
	**MRT_S *r (p)***	**MRT_M *r (p)***	**MRT_S *r (p)***	**MRT_M *r (p)***	**MRT_S *r (p)***	**MRT_M *r (p)***	**MRT_S *r (p)***	**MRT_M *r (p)***
FA	ASD	−0.04 (0.85)	0.17 (0.44)	−0.31 (0.39)	−0.09 (0.80)	−0.01 (0.98)	−0.05 (0.89)	−0.48 (0.03)^*^	−0.57 (0.01)^**^
	Con	−0.14 (0.53)	−0.33 (0.13)	0.12 (0.77)	−0.32 (0.39)	0.25 (0.43)	0.09 (0.79)	−0.01 (0.98)	0.02 (0.94)

(p, p value; r, Pearson correlation co-efficient; MRT_S, mean response time on Same trials; MRT_M, mean response time on Mirror trials).

* Indicates statistical significance p < 0.05;

** indicates statistical significance p < 0.01.

**Table 7:** **Results of correlation analysis between diffusion measures in white matter tracts and functional connectivity during Same and Mirror trials of a mental rotation task (*p*, *p*-value; *r*, Pearson correlation co-efficient; FC_S − Negative functional connectivity on Same trials; FC_S + Positive functional connectivity on Same trials, FC_M − Negative functional connectivity on Mirror trials, FC_M + Positive functional connectivity on Mirror trials, Con: Control, ^∧^1 Insufficient data for correlation as all functional connectivity values were negative between seed regions in left BA19 and left caudate head)**.

		**FC_S − *r (p)***	**FC_S + *r (p)***	**FC_M − *r (p)***	**FC_M + *r (p)***
**LEFT BA19 − LEFT CUNEUS**					
FA	ASD	0.09 (0.75)	−0.13 (0.81)	−0.10 (0.70)	0.01 (0.99)
	Con	−0.49 (0.06)	0.89 (0.11)	−0.01 (0.96)	^∧^1
		**FC_S −**	**FC_S +**	**FC_M −**	**FC_M +**
**LEFT BA19 − LEFT CAUDATE HEAD**					
FA	ASD	0.09 (0.82)	^∧^1	−0.08 (0.84)	^∧^1
	Con	0.31 (0.42)	^∧^1	0.31 (0.42)	^∧^1
**LEFT BA19 − LEFT THALAMUS**					
FA	ASD	0.23 (0.72)	−0.92 (0.01)^**^	0.40 (0.60)	0.53 (0.28)
	Con	0.69 (0.03)^*^	^∧^1	0.21 (0.55)	^∧^1
**LEFT BA19 − LEFT CAUDATE BODY**					
FA	ASD	0.30 (0.57)	0.16 (0.56)	0.09 (0.84)	−0.48 (0.04)^*^
	Con	0.36 (0.28)	−0.23 (0.54)	0.31 (0.33)	−0.53 (0.18)

* Indicates statistical significance p < 0.05;

** indicates statistical significance p < 0.01.

#### Correlation analyses of behavioral measures, diffusion measures, and functional connectivity in left BA19/left thalamus region

***Controls.*** In controls, there was a significant correlation between negative functional connectivity during Same trials, and MRT during these trials (*r* = 0.80, *p* < 0.006), indicating that a reduction in MRT (i.e., faster task performance) was associated with stronger negative connectivity (see Table 5 and see Figure 6).

There was no significant correlation between the diffusion measure of FA and MRT.

There were correlations between structural organization of the WM connecting the regions in left BA19 and left thalamus and functional connectivity between these regions. FA was significantly correlated (*r* = 0.69, *p* < 0.03) with negative functional connectivity on Same trials in controls (see Table 7 and see Figure 6). This correlation indicates that as WM organization increases (with increasing FA), there is a correlated reduction in the strength of negative functional connectivity.

***ASD.*** In the ASD group, there were no correlations between mental rotation performance (MRT) and functional connectivity or WM integrity of tracts between left BA19 and left thalamus (see Tables 5, 6 and see Figure 6). WM organization was significantly correlated with functional connectivity. FA was associated with positive functional connectivity in Same trials (FA *r* = −0.92, *p* < 0.01; see Table 7 and see Figure 6). These results indicate that a greater level of microstructural organization in the WM between left BA19 and left thalamus (increased FA) is associated with a reduction in positive functional connectivity.

#### Correlation analyses of behavioral measures, diffusion measures, and functional connectivity in left BA19/left caudate body region

***Controls.*** In controls, there were no correlations between the diffusion measure, functional connectivity, and MRT during mental rotation (see Tables 5–7 and see Figure 6).

***ASD.*** In the ASD group there was a significant correlation between the behavioral measure of MRT during Mirror trials and positive functional connectivity (*r* = 0.53, *p* < 0.02; see Table 5 and see Figure 6). This indicates that faster MRT on Mirror trials is associated with reduced strength of positive functional connectivity.

There was a significant correlation between FA and MRT on Same (*r* = −0.48, *p* < 0.03) and Mirror (*r* = −0.57, *p* < 0.01) trials indicating that as microstructural organization of the WM linking left BA19 and left caudate body increases (characterized by an increase in FA), there is an associated reduction in MRT (i.e., faster MRT) in the ASD group (see Table 6 and see Figure 6).

There was also correlation between FA and functional connectivity in the ASD group. FA was significantly correlated with positive functional connectivity on Mirror trials (*r* = −0.48, *p* < 0.04; see Table 7 and see Figure 6) indicating that increased structural organization of WM (increased FA) is associated with reduced strength of positive functional connectivity.

#### Correlation analyses of behavioral measures, diffusion measure, and functional connectivity in left BA19/left cuneus region

For both control and ASD groups, no significant correlations were found between the functional connectivity and MRT data (Table 5), the diffusion data and mean RTs (Table 6), or the diffusion data and functional connectivity data (Table 7).

#### Correlation analyses of behavioral measures, diffusion measure, and functional connectivity in left BA19/left uncus region

There were no significant correlations between the diffusion measure and behavior (see Table 6). Power was limited however, by the small sample size. There was insufficient data to perform correlation analyses of functional connectivity and diffusion measures or functional connectivity and behavioral measures.

## Discussion

The main finding of this study is that there are microstructural abnormalities in WM tracts that directly connect brain regions showing abnormal functional connectivity in participants with ASD. In addition, there are significant correlations between measures of WM microstructure, functional connectivity and behavior, which provide insight into the relationships between brain structure, brain function, and information processing in both neurotypical controls and individuals with ASD.

This discussion focuses on the implications these results have for the original hypotheses of this study, which predicted firstly that there would be WM tracts linking some, but not all pairs of brain regions showing abnormal functional connectivity, secondly that WM structure would be abnormal in tracts directly connecting the functionally defined regions and finally that there would be relationships between microstructural organization of WM, functional connectivity and behavior.

### Functional connectivity is not always associated with direct white matter connections

In this study, 10 pairs of brain regions were used as regions for selecting fiber pathways, reconstructed with CSD-based tractography. These regions were generated from functional connectivity maps during a mental rotation task, and indicated brain regions between which there was abnormal functional connectivity in ASD. Tractography analysis revealed that there were WM tracts directly connecting five of these 10 pairs of regions in most participants. For the other five region pairs there were no direct structural connections in any participants. This finding supports the first hypothesis. This finding of a direct structural connection between only half of the regions showing functional connectivity is consistent with results of imaging studies that have used a similar multimodal approach to integrate fcMRI and diffusion MRI. One of the first studies to use this approach reported that high functional and low structural connectivity can co-occur, but that low functional connectivity rarely occurs between regions where there is high structural connectivity (Koch et al., 2002). In keeping with this finding, a more recent study investigating the links between resting state functional connectivity and structural connectivity revealed that functional connectivity between regions is not indicative of a direct structural connection between those regions (Honey et al., 2009). This is likely to be because functional connectivity can be mediated by indirect connections or by input from a third region into the two regions, which modulates connectivity in the two primary regions (Koch et al., 2002; Honey et al., 2009; Behrens and Sporns, 2012).

### Abnormal functional connectivity is associated with abnormal structural connectivity

As discussed in section Evidence for a Relationship Between Brain White Matter Structure and Functional Connectivity in Neurotypical Populations of the introduction, previous studies have demonstrated a relationship between abnormal functional connectivity and abnormal structural connectivity (Quigley et al., 2003; Johnston et al., 2008; Lowe et al., 2008). Consistent with the prediction that abnormal functional connectivity would be associated with abnormal structural connectivity in the current study, there was reduced microstructural organization of WM in two of the five tracts linking regions of abnormal functional connectivity. In the ASD group, there was a significant reduction in the strength of functional connectivity between an occipital region (left BA19) and the left caudate head and also between this occipital seed region and the left thalamus. Analysis of diffusion measures in the WM directly linking these occipito-striatal and occipito-thalamic regions revealed significant microstructural abnormalities in the ASD group, which were characterized by reduced FA and CL, two measures that provide an indication of the level of organization of WM fibers. This finding of altered structural connectivity between brain regions that also show reduced functional connectivity is particularly interesting as it provides novel evidence to suggest that structural brain pathology may contribute to the abnormal functional connectivity that has been widely reported in the autism literature.

It is also noteworthy that the WM in both these tracts formed part of the left IFOF, a major WM association tract in the human brain. Interestingly, this study revealed structural abnormalities in this sub-region of the left IFOF, whereas a previous analysis of the whole left IFOF using the same data from the same study population found no abnormalities of WM (McGrath et al., 2013). This is of relevance as it supports a concern that current whole brain or even tract-specific analyses of WM may lack sensitivity in detecting WM abnormalities, and may not be specific enough about the exact locations of pathology in cases where abnormalities are reported.

It is important however to note that for the remaining three of the five WM tracts directly connecting regions showing abnormal functional connectivity, there was no evidence of disrupted organization of WM. In addition, in only one of these three tracts were there significant correlations between DTI and fcMRI measures (discussed in more detail in the following section). These findings are consistent with the theory that functional connectivity can be modulated by factors other than the level of microstructural organization of WM connecting brain regions. Such factors include the number of WM connections between regions; Hermudstat et al. recently demonstrated that the number of WM connections is positively correlated with the strength of resting state functional connectivity (Hermundstad et al., 2013). There are numerous diffusion measures that could be used to infer a level of “structural connectivity” in the human brain, but to date, the impact of most of these measures on functional connectivity is poorly understood. Neurochemical factors may also play an important role in modulation of functional connectivity, but a detailed discussion of these factors is outside the scope of this manuscript.

### Correlations between microstructural organization of white matter, functional connectivity and behavior

Correlation analysis revealed intriguing links between WM microstructure, functional connectivity and behavior in two of the five pairings in which there were direct WM tract connections; between regions in left BA19 and left thalamus, and between left BA19 and left caudate.

Between occipito-thalamic regions, functional connectivity was associated with behavior (faster visuospatial processing was associated with stronger negative functional connectivity) and with FA (stronger negative functional connectivity was associated with reduced microstructural organization) in the control group only. These correlation analyses suggest that during visuospatial processing, neurotypical controls benefit from increased functional inhibition between left BA19 and left thalamus, which is associated with reduced organization in WM between these regions. In the ASD group, structural and functional connectivity between occipito-thalamic regions were correlated (reduced microstructural organization was associated with reduced strength of positive functional connectivity). There were no statistically significant correlations between visuospatial processing speed and structural or functional connectivity, therefore the effect of altered connectivity in this tract on visuospatial processing is not known. It is interesting that both groups show a similar (statistically significant) relationship between structural and functional connectivity whereby less well-organized WM (reduced FA) is associated with increased functional suppression between regions. This may indicate that both ASD and control groups use this occipito-thalamic tract in a qualitatively similar way during visuospatial processing. This study has shown a reduction in FA in this tract in the ASD group. Given the correlation in the control group that shows a relationship between reduced FA and faster response times, it is possible that the reduced FA in the ASD group may contribute to their relative behavioral advantage in visuospatial processing.

Functional connectivity between left BA 19 and left caudate body was significantly increased in the ASD group relative to controls, but WM organization in the tracts directly connecting these regions was normal. The correlation analyses however implied that there were significant between-group differences in the functional use of this tract during visuospatial processing. Controls showed no association between structural, functional, and behavioral measures, whereas structural connectivity, functional connectivity, and visuospatial processing speed appeared to be strongly related in the ASD group. The lack of correlations in controls is in sharp contrast to the strong relationships found in the ASD group and might indicate that the ASD group relies on connectivity between these regions during visuospatial processing, whereas the controls do not. In relation to correlations observed in the ASD group, firstly greater organization of WM (higher FA) was associated with reduced functional connectivity between left BA19 and left caudate. Secondly, reduced functional connectivity between these regions was associated with faster MRT. Finally, faster MRT was correlated with greater microstructural organization of the WM between left BA19 and left caudate body. When considered together, these correlations suggest that a higher level of structural organization of this tract confers a benefit to visuospatial processing speed in ASD that may be mediated by increased functional suppression between left BA19 and left caudate. This finding is perplexing, as higher levels of WM organization have previously been associated with stronger rather than weaker functional connectivity; van de Heuvel reported a positive correlation between strength of functional connectivity in the default mode network and the level of FA in the cingulum (van den Heuvel et al., 2008) and another study demonstrated that increased radial diffusivity in WM connecting right and left primary sensorimotor cortices was associated with reduction of functional connectivity between these regions (Lowe et al., 2008). A recent paper specifically investigating the relationships between structural connectivity and resting state/task-based functional connectivity in the human brain' (Hermundstad et al., 2013), did not investigate the degree of organization of WM, but revealed that it is a high number of connections that facilitate strong resting state functional connectivity. In the current study, FA rather than number of connections was chosen as the measure of structural connectivity. In future studies investigating relationships between structural and functional connectivity, the measure(s) used to infer structural connectivity should be carefully considered.

Correlation analyses of connectivity and behavior between left BA19 and left caudate head and between left BA19 and left cuneus regions did not yield such strong evidence for an inter-relationship of structure, function, and behavior. There were no relationships between brain structure, functional connectivity or behavior. It is difficult to speculate on the reasons for this relative lack of structure/function/behavior correlations between these regions because, as discussed already, there is very little literature documenting relationships between brain WM structure and functional connectivity. There is however increasing recognition of the urgent need for research investigating links between anatomical connectivity, functional connectivity and behavior; this knowledge is crucial to understand the “capabilities of and constraints on human cognitive function” (Hermundstad et al., 2013).

### Direct implications of this study

Together, these findings offer a fascinating insight into the relationships between brain structure, brain function, and information processing in both neurotypical controls and individuals with ASD. This multimodal imaging study has used a novel approach to integrate functional and structural neuroimaging data. It has demonstrated, for the first time in ASD research that there is reduced microstructural organization of WM in tracts that directly connect brain regions that show abnormal functional connectivity. It also reveals that in some brain regions, individual differences in WM organisation are related to the level of functional connectivity during a visuospatial processing task, and further that this relationship has consequences on behavior.

There are many studies investigating functional or structural connectivity in ASD; however to date none have attempted to relate the two types of connectivity, an approach that is vital to increase understanding of the underlying neurobiology. The approach that is described in this study is rational and clinically feasible. It is hoped that future neuroimaging research in ASD will follow this type of methodology to integrate investigation of functional and structural connectivity. It will be interesting to see the impact of abnormal structural connectivity on functional connectivity during other neuropsychological paradigms and at rest.

### Potential implications of findings from current study on therapeutic interventions for autism

A greater understanding of the specific deficits in functional and anatomical connectivity in autism is particularly salient as there is some evidence to suggest that connectivity abnormalities are amenable to training interventions. Neuroplasticity in humans is well-documented (Doidge, 2007) and two fascinating studies have demonstrated training-related changes in brain WM structure (Keller and Just, 2009; Scholz et al., 2009). One study demonstrated that healthy adults who were trained on a complex visuo-spatial skill (juggling) developed an increase in FA in WM underlying the intraparietal sulcus (Scholz et al., 2009), while the other reported that after 100 h of intensive remedial instruction, children with impaired reading ability showed an increase in FA in a brain region that, prior to instruction, had showed significantly lower FA relative to good readers (Keller and Just, 2009). In addition, a recent study in patients with schizophrenia reported that improvement in brain functioning following cognitive remediation therapy might be based on an increase of the interhemispheric information transfer between the bilateral prefrontal cortexes via the corpus callosum (Penades et al., 2013).

That WM structure can be influenced by experience is highly relevant for autism research. WM integrity is abnormal in numerous regions in autism; but it may be possible to introduce therapeutic training to stimulate improvement in WM organization. Although this study focuses on visuospatial processing, a cognitive function that is enhanced in ASD, it has revealed abnormal WM in a number of discrete brain regions. It is crucial to characterize the WM deficits in ASD to develop targets for future treatments, which could conceivably focus on interventions that improve WM organization and inter-regional brain connectivity. Improved brain connectivity in ASD may lead to improvements in the behaviors that are often impaired in this condition.

### Limitations

There were a number of limitations to this study. Participants with ASD were limited to male, right-handed individuals with average or above-average IQ. Results are therefore very specific to this group and are not representative for all individuals on the spectrum. In this study, we adopted a novel approach whereby we specifically isolated WM tracts that directly connected brain regions showing abnormal functional connectivity. This approach was chosen as a primary aim of this research was to try to increase understanding of the neural correlates of atypical visuospatial processing in ASD and it was felt that a rational approach would be to use functionally defined ROIs from the connectivity analysis for diffusion tractography. This method allowed specific examination of the microstructural organization of WM in tracts that directly connected brain regions showing abnormal connectivity. It is important to point out however that there were a number of difficulties inherent in this approach. For example, it was not possible to back-project all functionally generated regions into native diffusion space and it is likely that valuable information about frontal and cerebellar WM abnormalities was not analysed in this study as a result. In addition, tracts between most of the fcMRI-defined ROIs are traceable in only a subset of participants. Also, we did not analyse any pairs of interhemispheric brain regions. It is also important to note that there are alternative approaches to investigating brain structural-functional connectivity relationships that were not adopted in the current study. For example one approach might be to define ROIs for tractography based on the location of between-group differences in diffusion measures, and move forward toward looking at the functional connectivity of the connected regions. In this study the diffusion measures used to infer structural connectivity were FA, CL, and CP. Recent research however has shown significant correlations between the number of tracts and functional connectivity, and researchers investigating relationships between functional and structural connectivity should carefully consider the measure(s) of “structural connectivity” selected.

In an attempt to increase understanding of the relationships between brain structural connectivity, functional connectivity and behavior, a large number of correlation analyses were carried out. Given the extremely limited data in both autism and healthy populations on the relationships between WM structure, functional connectivity and behavior, it was felt that it was reasonable to perform this number of exploratory correlation analyses; however, it is important to point out that correction for multiple comparisons was not carried out, thus some of the significant correlations reported may have been due to chance. In addition it is important to note that sample sizes for some of these correlation analyses were very small, which limited power. Nonetheless, it is illuminating to note the significant inter-relatedness of structure, function, and visuospatial processing speed in two of the WM tracts investigated (between the left occipital lobe and left caudate, and left occipital lobe and left thalamus). The correlations between these three measures lend strength to the hypothesis that there are indeed relationships, albeit complex, between structure, function, and behavior.

### Conclusion

This novel multimodal imaging study has identified aberrant WM microstructure in tracts that directly connect brain regions that are abnormally functionally connected during visuospatial processing in ASD. Exploratory correlation analyses have revealed associations between structural connectivity, functional connectivity and visuospatial processing speed in both ASD and control groups, however the dearth of literature on normal relationships between DTI, fcMRI, and behavior makes it difficult to speculate on the true meaning of these associations. There is an urgent need for further research investigating links between structural connectivity, functional connectivity and behavior in both neurotypical and ASD populations. It is critical to understand the complex neural pathophysiology of autism in order to develop rational, targeted therapeutic interventions to improve WM organization and inter-regional neural connectivity.

### Conflict of interest statement

The authors declare that the research was conducted in the absence of any commercial or financial relationships that could be construed as a potential conflict of interest.
